# Enhancement of Cell Membrane Invaginations, Vesiculation and Uptake of Macromolecules by Protonation of the Cell Surface

**DOI:** 10.1371/journal.pone.0035204

**Published:** 2012-04-30

**Authors:** Nadav Ben-Dov, Rafi Korenstein

**Affiliations:** Department of Physiology and Pharmacology, Faculty of Medicine, Tel-Aviv University, Tel-Aviv, Israel; University of Cambridge, United Kingdom

## Abstract

The different pathways of endocytosis share an initial step involving local inward curvature of the cell’s lipid bilayer. It has been shown that to generate membrane curvature, proteins or lipids enforce transversal asymmetry of the plasma membrane. Thus it emerges as a general phenomenon that transversal membrane asymmetry is the common required element for the formation of membrane curvature. The present study demonstrates that elevating proton concentration at the cell surface stimulates the formation of membrane invaginations and vesiculation accompanied by efficient uptake of macromolecules (Dextran-FITC, 70 kD), relative to the constitutive one. The insensitivity of proton induced uptake to inhibiting treatments and agents of the known endocytic pathways suggests the entry of macromolecules to proceeds via a yet undefined route. This is in line with the fact that neither ATP depletion, nor the lowering of temperature, abolishes the uptake process. In addition, fusion mechanism such as associated with low pH uptake of toxins and viral proteins can be disregarded by employing the polysaccharide dextran as the uptake molecule. The proton induced uptake increases linearly in the extracellular pH range of 6.5 to 4.5, and possesses a steep increase at the range of 4> pH>3, reaching a plateau at pH≤3. The kinetics of the uptake implies that the induced vesicles release their content to the cytosol and undergo rapid recycling to the plasma membrane. We suggest that protonation of the cell’s surface induces local charge asymmetries across the cell membrane bilayer, inducing inward curvature of the cell membrane and consequent vesiculation and uptake.

## Introduction

One of the basic functional characteristics of the cell plasma membrane is its ability to facilitate organized and controlled uptake of molecules from the extracellular milieu and participate in signaling processes (for a review see [1]). While essential small molecules, such as amino acids, sugars and ions, can traverse the plasma membrane through the action of membrane transporters or channels, macromolecules must be carried into cells by endocytosis. Endocytosis utilizes multiple endocytic pathways for different types of cargo subdivided into clathrin-dependent and clathrin-independent routes [1]. However, the different pathways of endocytosis share an initial step involving local inward curvature change of the cell’s lipid bilayer [2]. This curvature change is followed by the formation of different shapes of invaginations in the plasma membrane, for different pathways, where the curvature of the membrane is extensively increased, upon the creation of spherical, ellipsoid or tubular structures. Consequently there is a formation of a narrow membrane neck that undergoes scission, leading to the release of the vesicular structure into the intracellular milieu. The existence of clathrin- and caveolin-independent forms of endocytosis raises the question of how these vesicles are actually formed. The necessity to deform membranes, especially to a high degree of curvature observed in cells, is in stark contrast with the tendency of lipid bilayers to be planar. In recent years the role of proteins and lipids in generating and sensing membrane curvature has gained better understanding (for recent reviews see [2], [3], [4]. It has been shown that to generate membrane curvature, proteins can either make the lipid bilayer asymmetric with respect to its mid plane, or apply forces or mechanical constraints (scaffolds) to the membrane surface forcing the membrane to bend [3]. This is accomplished by a direct insertion of protein domains into the membrane monolayers changing the structure of one of them with respect to the other or by modifying the lipid compositions of the membrane monolayers in such a way that the monolayers become different in terms of either the total amounts of the lipid molecules, or concentrations of diverse lipid species, or both [5]. Thus, it emerges as a general phenomenon that transversal membrane asymmetry is the common required element for the formation of membrane curvature.

Other, non-endocytic pathways, which enable macromolecules to transverse the plasma membrane was evolved, most notably by pathogens and viruses. Cell penetrating peptides (CPPs) are amphiphilic peptides of up to 30 amino acids, which can be internalized into cells by mechanisms that may not require cellular energy. The two common features of all CPPs appear to be a positive charge and amphipathicity. For example, the translocation of peptide with an α-helical structure, could be associated with membrane pore formation [6], [7]. CPP’s affect membranes of cells and organelles, resulting from the specific interaction of CPPs with cell components [for review see [8]].

A wide variety of toxins and virus enveloped proteins take advantage of the low pH in the endocytic pathway to facilitate endosomal escape. Direct entry from the cell surface can be induced likewise by exposing the cells to surface-bound toxin or viruses at low pH, thereby mimicking the conditions in the endosomes [9]. One study also suggested that other, non-pathogenic proteins may also enter the cells on the basis of similar mechanisms [10]. It had been theoretically suggested that in pH-dependent fusion proteins, critical histidine is located close to positively charged residues, possibly interacting via hydrogen bonds. At low pH, histidine becomes positively charged, disrupting any existing hydrogen bonds and leading to electrostatic repulsion. Upon protein refolding, histidine would form a salt bridge with a negatively charged residue, thus stabilizing the post-fusion conformation [11]. The critical role of histidine in pH dependent membrane fusion has been demonstrated by point mutation of histidine residues in viral fusion proteins [12], [13]. It is now accepted that entry of virus into host cells through endosomal escape pathway is controlled by two envelope glycoproteins, E1 and E2 [14]. Low pH dissociates the E2/E1 dimer, releasing the membrane fusion protein E1 that refolds into a trimeric hairpin conformation, thus driving the fusion reaction [15].

The present study describes a new, yet unidentified route, for uptake of proteins and non-protein molecules, driven by cell exposure to a high proton concentration. We present data showing that proton-induced uptake is independent of endocytosis pathways and unrelated to the low pH triggered fusion mechanism by which some proteins and peptides can transverse the cell membrane. We suggest that acidification of the cell surface is sufficient to induce inward invaginations of the membrane with the consequent fission of vesicles, leading to an increased uptake of macromolecules into the cytoplasm.

## Results

### Exposure of Cells to External Low pH Induces Inward Budding of the Plasma Membrane

**Figure 1:**
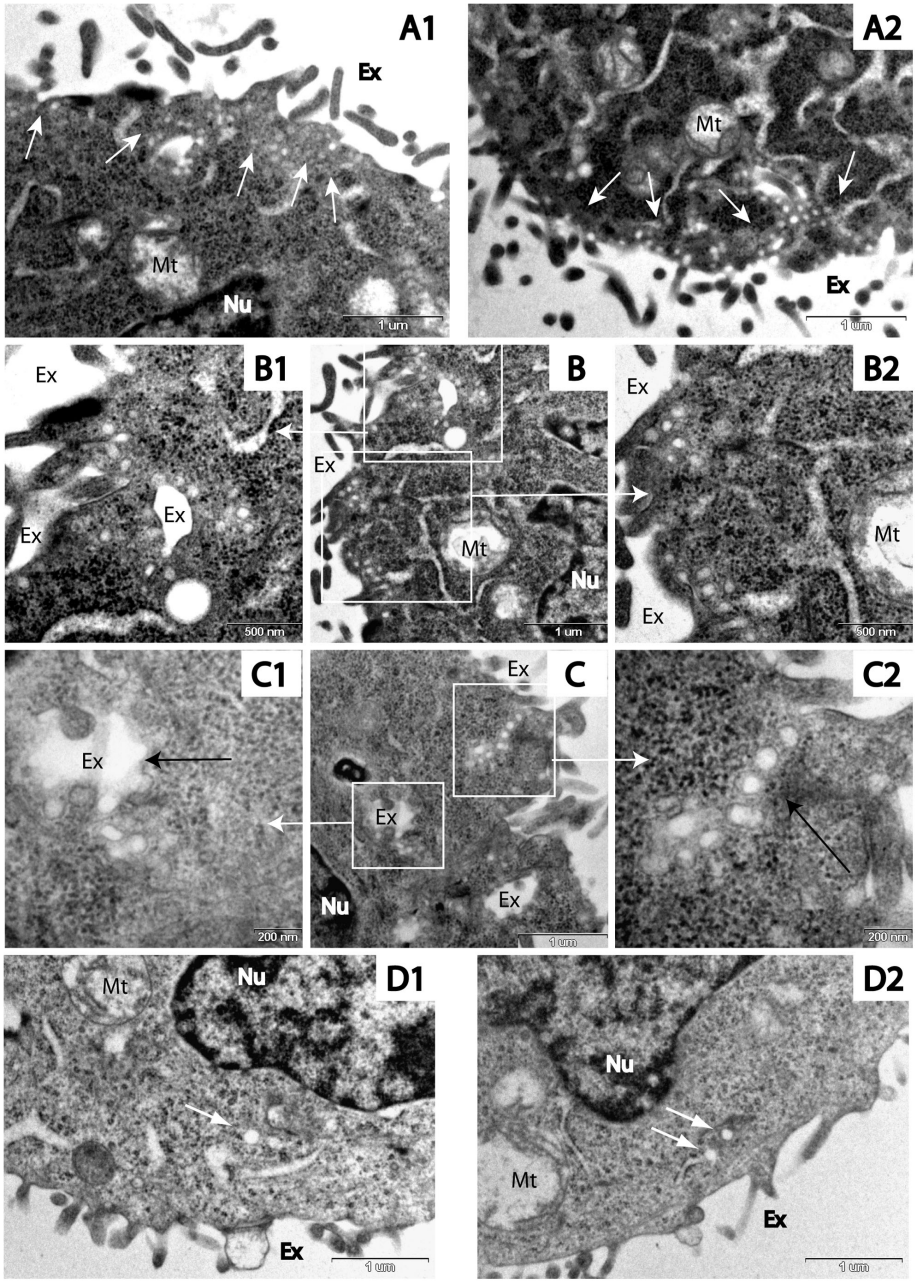
TEM images of cells during exposure to low pH. HaCaT cells cross-sections, fixed during exposure to pH 5, reveal distinct areas of high prevalence of vesicular structures (see white arrows in A-1 and A-2, bar size = 1 µm). Cell cross-section populated with vesicular structures (B, bar size = 1 µm), is enlarged in B-1 and B-2 (bar size = 500 nm). Cell cross section (C, bar size = 1 µm) containing large membrane enclosure (black arrow in C-1, bar size = 200 nm) coupled with buds and vesicles; a chain of vesicles (black arrow in C-2, bar size = 200 nm). Control cells fixed under pH 7.4 (D-1 and D-2, bar size = 1 µm). While no caveolae-like structures could be seen in these cells, some endosome-like vesicles are found (white arrows). Ex – Extracellular space; Nu – nucleus; Mt – Mitochondria.

Using transmission electron microscopy (TEM) visualization, we demonstrate that exposing cells to low external pH enhances the inward formation of plasma membrane budding. Cross-sections of the HaCaT cells that were fixed immediately following their exposure to external pH 5, reveal distinct areas in the vicinity of the plasma membrane which are populated with vesicular structures (Fig.1-A) that possess diverse morphologies (Fig. 1-B and 1-C). In cells that were fixed for 15 min after the medium pH was restored to pH 7.4, we could not detect any of these characteristic membrane structures. Control cells exposed to physiological pH 7.4 reveal no vesicular structures of similar scale and abundance (Fig. 1-D).

**Figure 2:**
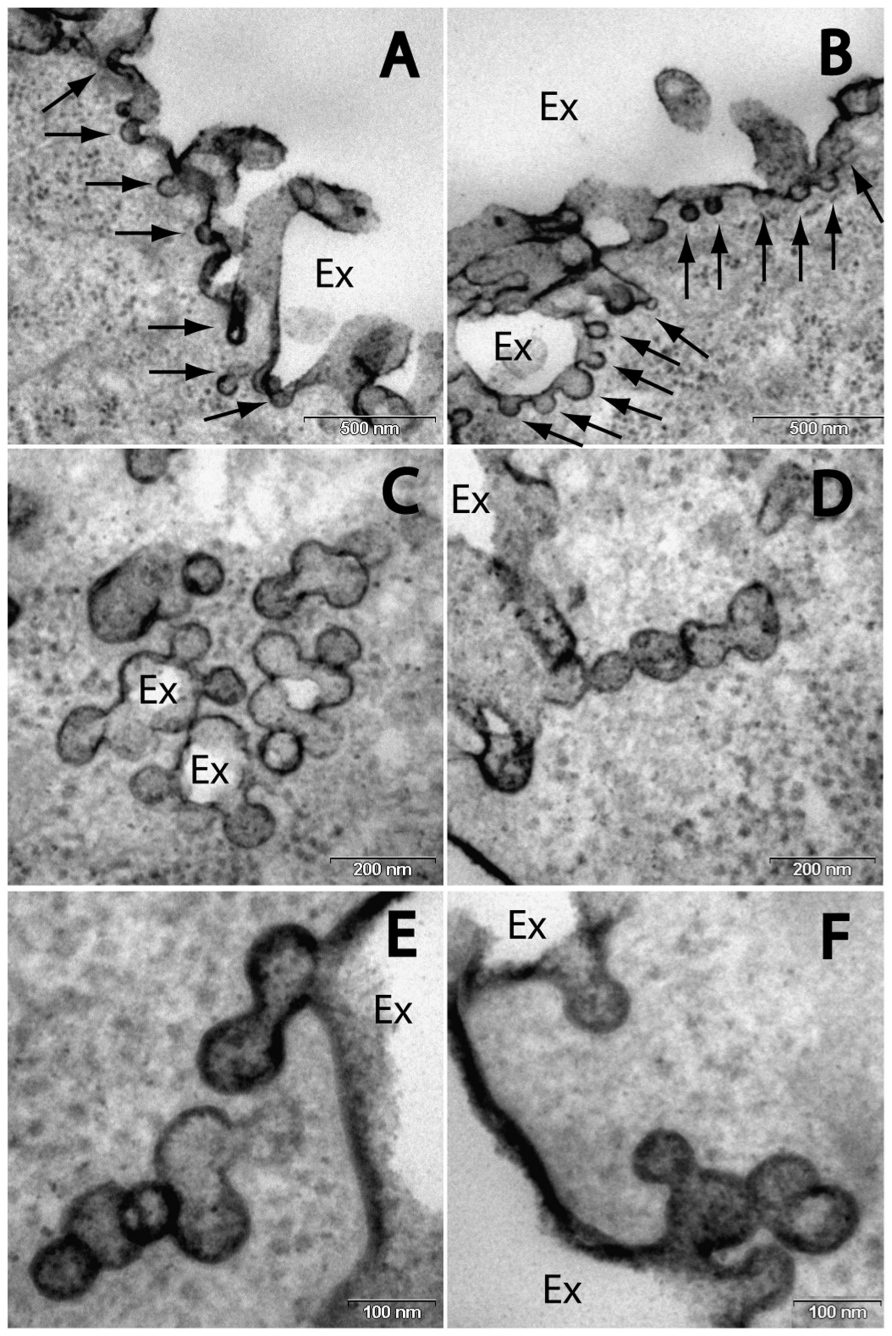
Images of cells during exposure to low pH with the plasma membrane labeled by tannic acid. Areas of the plasma membrane comprising high incidence of inward budding (A and B, bar size = 500 nm). Plasma membrane invaginations exist in different morphologies such as rosettes (C) or chains (D) (bar size = 200 nm). Higher magnification of membrane buds in intricate arrangements (E and F, bar size = 100 nm). Ex – Extracellular space.

In order to gain further insight as to the nature of these vesicular structures, the cells were fixed in the presence of 1% tannic acid (TA), which labels only the external surface of plasma membrane [16]. Thin sections of these cells were stained with lead citrate only and visualized by TEM. Fig. 2 depicts well-defined membrane structures that are in continuous connection with the plasma membrane. These structures appear in the form of buds (Fig. 2-A and 2-B), large irregular enclosures (“rosettes”, Fig. 2-C), chains (Fig. 2-D) and intricate formations (Fig. 2-E and 2-F).

**Figure 3:**
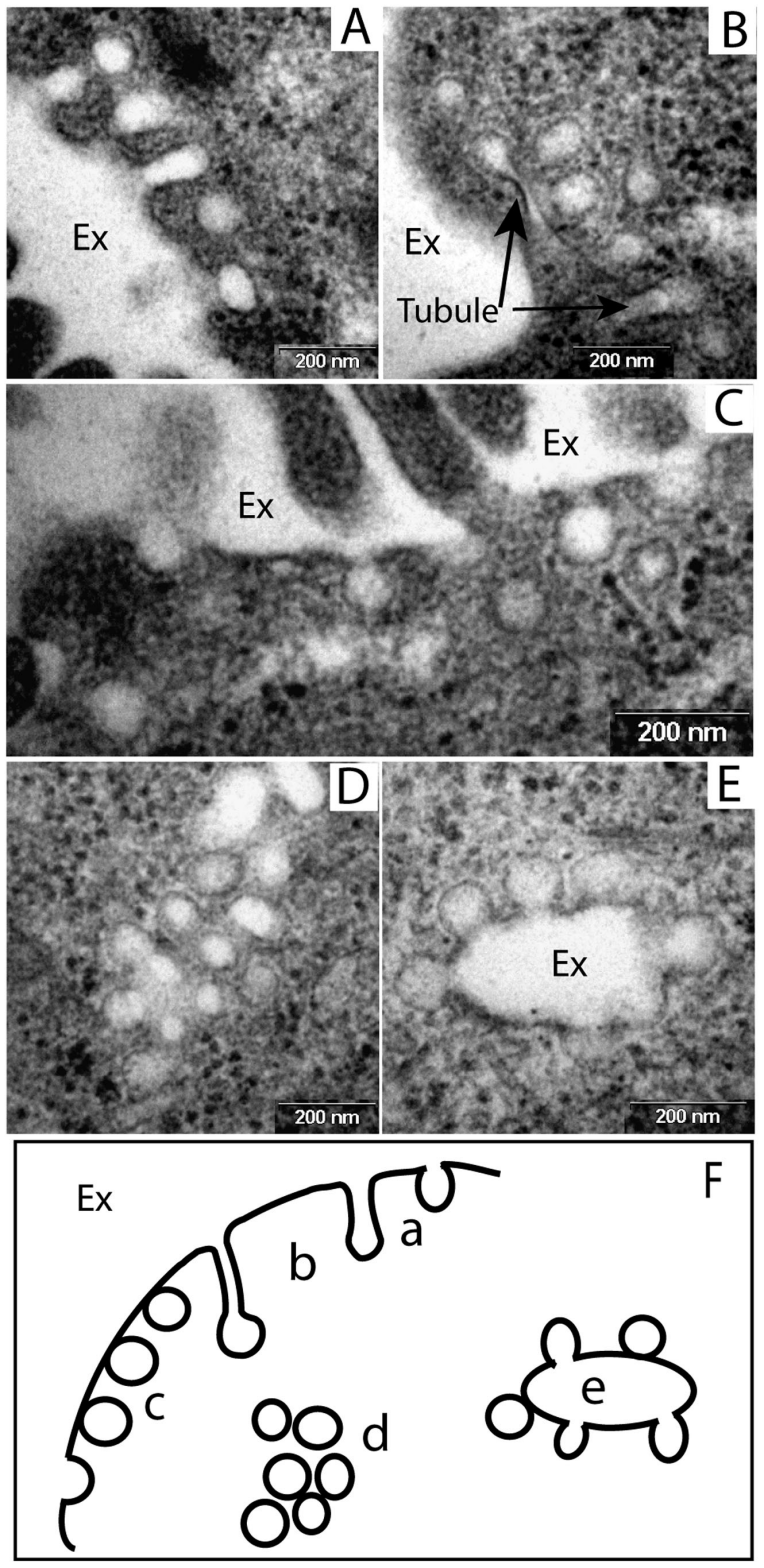
Different morphologies of membranous structures induced by low pH. TEM images from cells exposed to external low pH (A to E, bar size = 200 nm). (F) Schematic representation of membranous structures of different morphologies: plasma membrane buds (a), tubules (b), vesicles connected to the plasma membrane (c), clusters of membrane vesicles (d) and inward protrusions of the plasma membrane with buds and connected vesicles (e). Ex – Extracellular space.

The exposure of cells to external low pH is shown to trigger the formation of inward budding of the plasma membrane which disappears upon restoration of physiological pH level. These membrane structures can be classified into three groups (Fig. 3). Buds, tubules and connected vesicles are all subsets of structures which retain the connection to the plasma membrane (Fig. 3-A, 3-B and 3-C). Vesicles departed from the plasma membrane can appear in dispersed or clustered formations (Fig. 3-C and 3-D). Finally, the large enclosures “rosettes” are probably inward protrusions of the plasma membrane which can be associated with all other classes of membrane structures (Fig. 3-E). The nature of the round small circles (∼50 to 80 nm diameters) is less defined since they could be either the cross sections of budding tubules or represent vesicles already detached from the plasma membrane. As evidenced from Figs. 1 and 3, some of the small membrane circular structures are not accompanied by the larger irregular membrane enclosures and possess uniform size and morphology. These structures are not visible by TEM in cells stained with TA (Fig. 2), as compared with those stained by the standard Lead citrate and Uranyl acetate protocols (Figs. 1 and Fig. 3). As the TA is adsorbed only to those membrane sections that are in continuous connection to the plasma membrane, we suggest that that the circular structures in the vicinity of the plasma membrane represent intracellular vesicles rather than invaginations of the plasma membrane.

### External Low pH Enhances Uptake

The formation of inward membrane buds that can evolve into membrane vesicles, can potentially lead to the uptake of molecules into the cells. We suggest that the plasma membrane buds form an entry pathway for otherwise impermeable molecular entities. The possibility that enhanced uptake of molecules can take place due to the cell’s exposure to external low pH was also studied by a combination of fluorescent microscopy and flow cytometry.

Measurements of the intracellular pH, using the pH sensitive fluorescent probe BCECF shows that HaCaT cells, suspended in an external solution of pH 5.5, had a cytosolic pH of 6.5 which they were able to preserve for at least 60 min (Fig. S1). The cells regained their original cytoplasmic pH within less then 5 min upon buffering the external solution to pH 7.4. However, when the extracellular pH dropped down to 4.2, the cytosolic pH declined linearly.

**Figure 4:**
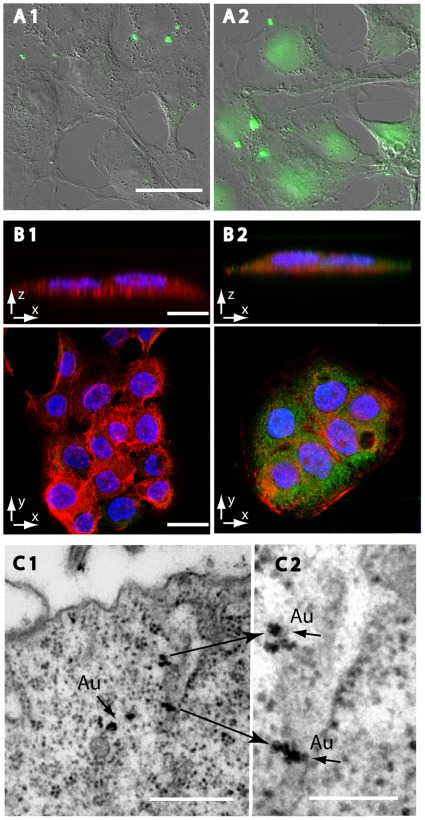
Cellular uptake of macromolecules following exposure to low pH. (A) Fluorescent images of HaCaT cells that were incubated with 10 µM dextran-FITC at either pH 7.4 (A1) or pH 5.25 (A2) at RT (bar size = 20 µm). The cells were incubated for 15 minutes in DMEM before being imaged. (B) SCLM images of HaCaT cells that were incubated with unspecific IgG-Cy5 (pseudo-colored in green) for 30 minutes at either pH 7.4 (B1) or pH 5.25 (B2). The cells were fixed with 4% paraformaldehyde and stained with DAPI (colored blue) and phalloidin-TRITC (colored red). Bar size for X–Z = 10 µm and for X–Y = 25 µm. (C) TEM images depicting gold particles (Au) in HaCaT cells that were incubated with unspecific IgG-Gold for 10 minutes at pH 5.25 and then fixed with karnovsky solution. C-2 (bar size = 200 nm) is a magnified section of C-1 (bar size = 500 nm).

HaCaT cell cultures were exposed to low pH 5.25 for 15 min in the presence of 43 kD dextran-FITC (10 µM) according to protocol and their images were acquired using both fluorescent (Ex 480 nm/Em 530 nm) and DIC microscopy and are presented as merged images (Fig. 4-A). The images reveal that dextran-FITC intensity is much lower at the thinner edges of the cells, suggesting correlation between the observed fluorescence intensity and cell thickness. Such correlation would not be possible if the dextran probe was merely adsorbed to the cell surface. In additional study, HaCaT cell cultures were exposed to pH 5.25 for 30 minutes in the presence of a non-specific immunoglobulin (IgG-Cy5, unspecific polyclonal sheep anti mouse). Cell images were captured by SCLM cross-sectioning and its reconstructions are presented at the X-Z and X-Y planes (Fig. 4-B). Viewing the cell’s cross sections, both from the X-Y and X-Z planes, reveals that the fluorescent probes are located inside the cell. We further extended our studies by exposing HaCaT cells to pH 5.25 as before, in the presence of Gold labeled-IgG (IgG-Gold, unspecific polyclonal goat-anti mouse) and prepared them for TEM analysis. Fig. 4-C demonstrates that gold particles are found in the cells’ cytoplasm.

**Figure 5:**
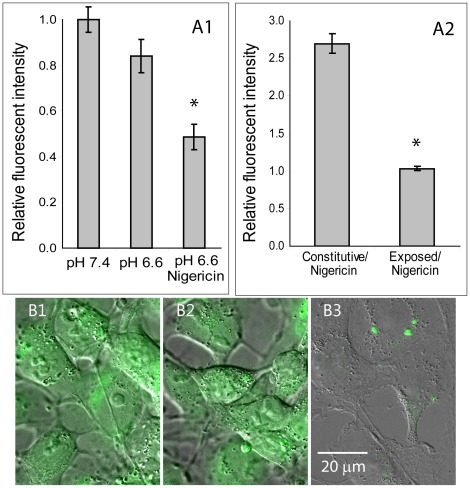
Determination of adsorbed VS internalized fraction of Dextran-FITC following uptake induced by low pH. (A1) HaCaT cells exposed to pH 5.25 in the presence of dextran-FITC were washed with K^+^PBS, and were analyzed three times by FACS, first at pH 7.4, second at pH 6.6 and third in the presence of 10 µM nigericin. The cells’ fluorescence intensities in the second and third analyses are presented as fraction of the first analysis. *P<0.05, n = 9) (A2) HaCaT cells exposed to pH 5.25 in the presence of dextran-FITC were washed with K^+^PBS, and were analyzed twice by FACS; first time at pH 7.4 and second time in the presence of 10 µM nigericin. The cells’ fluorescence intensities in the second analysis (in the presence of nigericin) are presented relative to the first analysis. *P<0.05, n = 9) (B) Microscopic images were acquired in the fluorescence (Em 530 nm) and DIC channels at X100 magnification. COS-7 cells were grown on glass bottom 96 wellplate and exposed to a solution of HBSS at pH 5.25 in the presence of 70 kD dextran-FITC (5 µM) for 15 minutes period, followed by washing with K^+^PBS at pH 7.4. (B1) Cultures incubated in K^+^PBS at pH 7.4. (B2) Cultures incubated in K^+^PBS at pH 6.0 (B3) Cultures incubated in K^+^PBS at pH 6 with 10 µM Nigiricin.

It is difficult to provide a quantitative estimation of the localization of fluorescent macromolecules, especially in the vicinity of the inward side of the cell membrane, by confocal microscopy due to the diffraction limited resolution. Therefore we employed a different approach in which the fraction of dextran-FITC adsorbed to the plasma membrane was estimated through the attenuation of FITC fluorescence by low pH. Cells were exposed to pH 5.25 in the presence of 70 kD dextran-FITC (5 µM) for 10 min before they were harvested and suspended in K^+^PBS. Flow cytometry analysis of the cells was first conducted with the cells suspended in K^+^PBS at pH 7.4. Immediately thereafter, the pH of the cell suspension was decreased to 6.6 (by HCl titration) and the suspended cells were analyzed for the second time by flow cytometry. Next, 10 µM nigericin (H^+^/K^+^ ionophore) was added to the cell suspension for 5 minutes at room temperature (RT), followed by a third flow cytometric analysis. For control, the analyses were repeated with the cells at pH 7.4, at all three stages. Fig. 5-A1 details the cellular fluorescence from the second and third analyses, compared to the fluorescence obtained from the first analysis. Lowering the extracellular pH alone had no significant effect on the cellular FITC intensity (P>0.05), suggesting that the FITC molecules are protected from the attenuating effect of the low pH. However, when the intracellular pH was lowered by the presence of nigericin, the cell’s fluorescent intensity dropped by 43% (P<0.05). Cells that were kept under pH 7.4 at all three stages had no significant attenuation of their fluorescence as a result of repeated handling and exposure to nigericin (P>0.05, by *t*-test). A visual confirmation of the flow cytometric results were obtained by fluorescent microscopy (Fig. 5-B1 to B3). These findings imply that no significant amount of dextran is adsorbed to the cell surface. As an additional precaution we added to our cell suspensions 0.01% trypan-blue (TB), an impermeable fluid-phase dye that quenches FITC fluorescence by direct collision energy transfer, during our flow cytometric analyses [17].

### Uptake Induced by Low pH is Independent of the Known Endocytosis Pathways

We have shown that triggering inward budding of the plasma membrane by low pH, increases the uptake of molecules located in the vicinity of the cell surface. The possibility that this uptake proceeds through the better known pathways of clathrin or caveolin dependent endocytosis or by macropinocytosis, was addressed by several avenues.

Nigericin was used with similar methodology as in the above section for evaluating the possibility that the uptake of dextran involves its entrapment in acidic endosomes. HaCaT cells were exposed to pH 5.25 in the presence of 70 kDa dextran-FITC (5 µM) for 10 min, thoroughly washed and suspended in K^+^PBS. For control, cells were treated under the same experimental procedure but at pH 7.4, representing the level of constitutive dextran uptake. 30 min after the termination of the treatment, the cell suspension was first analyzed by flow cytometry at pH 7.4. Following the addition of 10 µM nigericin, the cell suspension was analyzed for the second time. Comparing the fluorescent intensities of the two analyses (Fig. 5-A2) reveals that in cells of the control group, the addition of nigericin increased FITC fluorescence intensity by 2.5 folds (P<0.01, by two-tail t-test), as is expected when endosomal pH is balanced to that of the cytosol. In cells exposed to dextran-FITC at pH 5.25, the pH clumping by nigericin yielded no significant change in cellular fluorescence (P>0.05, by two-tail t-test), suggesting that dextran-FITC was not trapped inside acidic endosomes.

We have used the uptake of dextran-FITC as a functional assay to explore the dependence of proton induced uptake on the better known pathways that underlie classical endocytosis. Cultures of Caco-2/TC7 or HaCaT cells were pre-incubated for 15 min with known inhibitors, including 9 µM dingo (dynamin GTPase inhibitor [18]), 35 µM filipin (polyene antibiotic that sequester membrane cholesterol [19]), 200 µM genistein (Tyr phosphatase inhibitor [20]) 4 µM wortmannin (phosphatase inhibitor of IPK3 [21], [22]), and 50 nM calyculin-A (Ser/Thr phosphatase inhibitor [23]). Additionally we pre-incubated the cells with 10 mM Methyl-β-cyclodextrin for 30 min to deplete cholesterol from the plasma membrane [24]. The validation of these inhibitors’ competence on receptor mediated endocytosis was verified by testing their attenuating effect on the uptake of transferrin-Alexa 635 conjugate. The cells were then exposed to external pH 5.25 for 5 min in the presence of 5 µM dextran-FITC (70kD) according to protocol carried out at 24°C and their fluorescence analyzed by flow cytometry. None of the chemical agents employed had reduced the extent of uptake relative to untreated cells (P>0.1, by t-test with n = 9 for each inhibiting procedure).

Previous reports [25], [26], [27], [28], [29] have shown that below 10°C, endocytosis is negligible, yet when exposing the cells to external pH 5.25 at 4°C, the dextran-FITC uptake is 4.7 folds higher then the constitutive level, though 4 times lower than uptake performed at 24°C (both at P<0.05, t-test n = 9). It has also been reported [27], [28] that endocytosis is inhibited under depletion of energy resources. However, cells that had undergone ATP depletion by 95% before being exposed to external pH 5.25, yielded significantly higher dextran-FITC uptake then cells possessing normal ATP content under the same external low pH (P<0.01 by t-test, n = 9). Furthermore, lowering the external pH to 5.25 is accompanied by moderate acidification of the cytoplasm (to pH 6.5, see methods), a condition known by itself to prevent clathrin mediated endocytosis [30].

Our studies clearly show that subjecting the cells to conditions (e.g. low temperature, ATP restriction) or pharmacological agents that are known to inhibit endocytosis, has no attenuating effect on the extent of uptake mediated by low pH. In addition, following uptake, the dextran-FITC molecules were not confined to acidic endosomes, but located at sites possessing a pH typical for the cytoplasm. Taking into account the observed induction of cell membrane budding and the independency of the ensued macromolecules uptake from common endocytic pathways, we propose the term pH Induced Uptake, PIU, to describe this phenomenon.

### The PIU of Dextran-FITC is not Carried through Increased Membrane Permeability

The possibility that the fluorescent probe diffuses into the cytoplasm through pH-induced permeability of the plasma membrane was examined in flow cytometry analyses by employing two methods for addressing efflux or influx of small molecules during the exposure to low extracellular pH. In the first approach, cells were pre-loaded with the fluorescent probe BCECF-AM (Ex 485 nm/Em 530 nm) before being exposed to pH 5.25 or pH 7.4 for 30 min after which the cells were washed and maintained in cold PBS (pH 7.4). Allowing sufficient time to restore normal cellular pH, the possible attenuation of the cell’s fluorescence was monitored by FACS as an indication for the probe’s leak out. The difference in BCECF fluorescence between the cells exposed to low pH and control untreated cells, was statistically non significant (P>0.05, by *t*-test, n = 9,). In the second approach, the cells were exposed to pH 5.25 or pH 7.4 for 30 min in the presence of the fluid-phase nuclear florescent marker PI (2 µg/ml), after which the cells were washed and maintained in cold PBS (pH 7.4). PI can only diffuse into cells with compromised membranes, thus the possible augmentation of the cell’s fluorescence would have been an indication for the probe’s leak into the cell. The fraction of PI stained cells, as determined by FACS, is ∼ 5% in both control and experimental groups with no significant difference (P>0.05, by two-tail *t*-test, n = 12).

As both of these approaches did not yield statistically significant change in intracellular fluorescence, the possibility for uptake of larger macromolecule (i.e. 70 kD dextran-FITC) through low pH induced membrane permeability can be disregarded.

### Dependence of PIU on the Level of External pH

**Figure 6:**
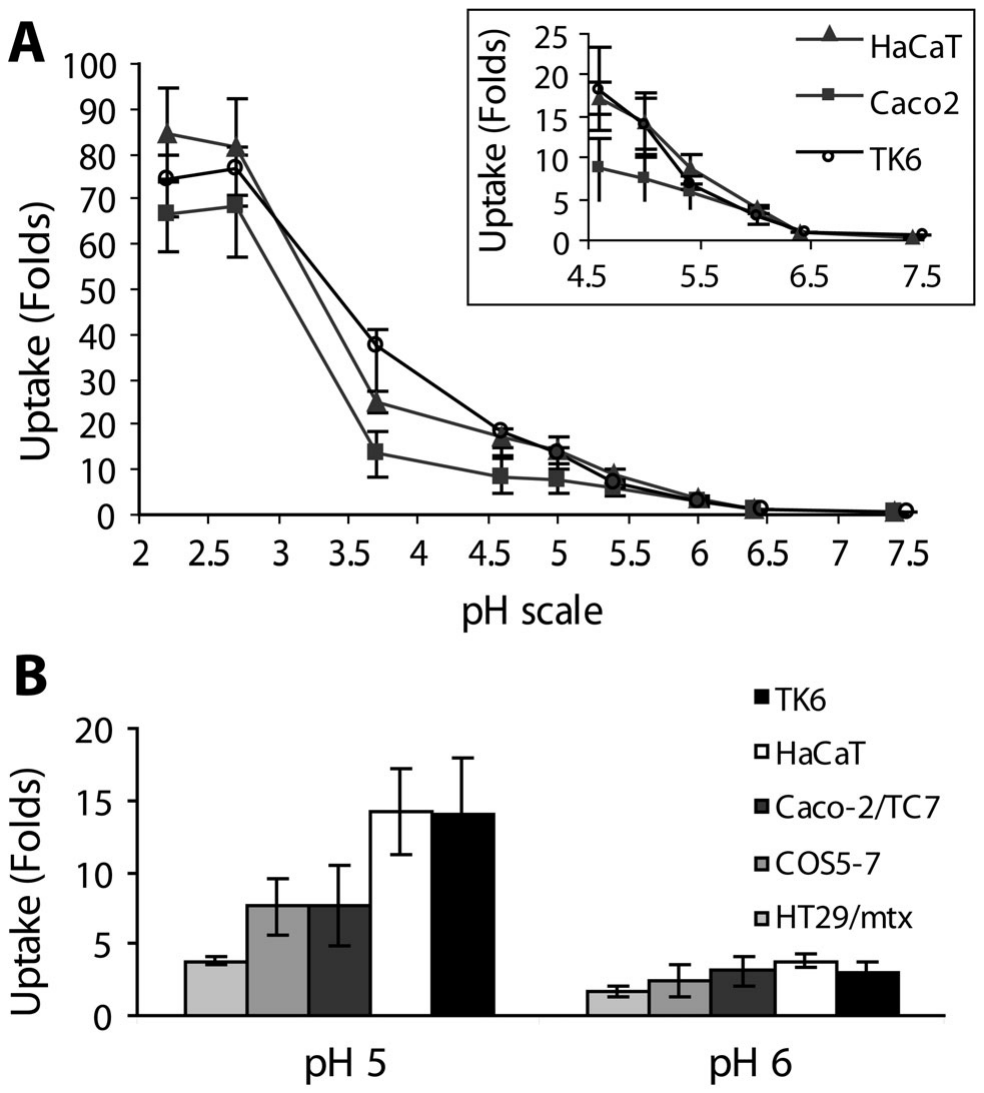
Dependence of PIU on extracellular pH. Adherent cell cultures (HaCaT, Caco-2/TC7, COS-7 and HT29/mtx) and non-adherent cultures (TK6) were exposed to solutions of different pH in the presence of dextran-FITC for a period of 5 minutes, before being washed, harvested and analyzed. Uptake, based on flow cytometry, is plotted as function of the external pH from 3 independent experiments per cell line (A) Extent of uptake in terms of fold of uptake (mean ± SD) relative to the constitutive uptake at physiological pH 7.4 (n = 9 for each cell line). (B) Fold of uptake (mean ± SD) relative to the constitutive uptake at physiological pH 7.4 (n = 9 for each cell line). At pH 6, the difference in intracellular dextran concentration among the cell lines is of border line significance (P = 0.056 by one way ANOVA). At pH 5, HaCaT and TK6 cells have 2 folds higher dextran concentration then Caco2/TC7 and COS-7 (P<0.001, *t*-test), which in turn have 2 folds higher dextran concentration than HT29 cells (P<0.001, *t*-test).

The dependence of PIU on external pH was monitored by measuring the uptake of dextran-FITC (5 µM, 70 kD). Fig. 6-A portrays PIU for three cell lines of different origin: adherent intestine endothelial (Caco-2/TC7) and skin epithelial (HaCaT) cell lines and a non-adherent lymphoblast line (TK6). The data shows that exposing the cells to external low pH for 5 min produces statistically significant uptake of dextran-FITC for pH≤6.5. This uptake increases linearly in the pH range of 6.5 to 4.5, and possesses a steep increase at the range of 4> pH>3, reaching a plateau at pH≤3. A similar dependence of cellular uptake on extracellular pH was found with other cell lines, including fibroblast COS-7 and the mucus-secreting HT29-MTX goblet cells (Fig. 6-B). While at external pH 6 the uptake of dextran-FITC by all cell lines shows little difference, at external pH 5 the uptake of Caco-2/TC7 and COS-7 is 2 folds higher than that of HT29-MTX and lower by 2 fold then that of TK6 and HaCaT. The relative high extent of PIU in TK6 cells compared to other cell lines could perhaps be attributed to the higher exposure of their cell surface to the extracellular fluid as compared with adherent cells. Among the adherent cell lines, the relative higher extent of PIU in HaCaT cells can perhaps be ascribed to their lower level of constitutive endocytosis and efflux pumps (MDR) activities [31], as compared with those of Caco2/TC7 and COS-7 cell lines [32]. The attenuation in uptake extent found for HT29-MTX cells is most probably attributed to the additional diffusion barrier of the mucus layer between the cells and the fluorescent probe in the external medium [33]. Thus PIU prevalence was validated in four different adherent cell lines and in a non-adherent cell line, indicating that this phenomenon is not restricted to a specific cellular model.

### Kinetic Profile of PIU

**Figure 7:**
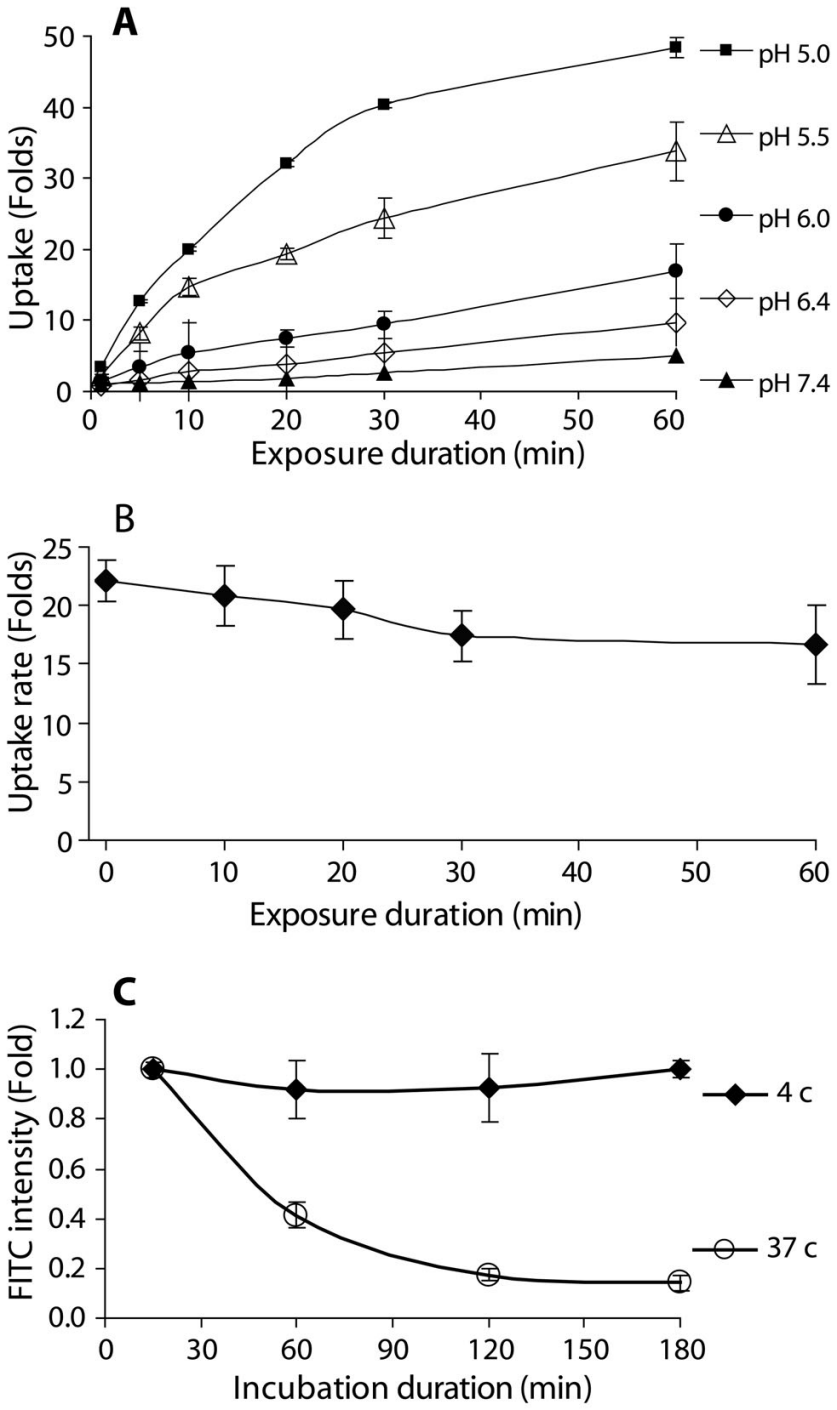
Kinetic profile of PIU. (A) The relative intensities of dextran-FITC (70 kD, 10 µM) in HaCaT cells are presented as function of external pH and duration of exposure. Suspensions of cells were exposed to solutions of varying pH (7.4, 6.4, 6, 5.5 and 5) in the presence of dextran-FITC for 6 time periods (1, 5, 10, 20, 30 and 60 min). Results from FACS analyses are presented as fold induction relative to the constitutive uptake at physiological pH 7.4, in terms of geometrical mean±SD (n = 15 per each pH level). (B) The rate of dextran PIU is presented as function of the time the cells were exposed to low pH. Rate was determined by introducing Dextran-FITC as a short pulse for the last 10 min of the cells’ exposure to pH 5.25. Results from FACS analyses are presented as fold induction relative to the constitutive uptake at physiological pH 7.4, in terms of geometrical mean±SD (n = 9). (C) The relative attenuation of the intracellular intensity of dextran-FITC, as function of incubation time and temperature. The cells were initially loaded with Dextran-FITC by mean of PIU. The time dependent exponential decrease in cellular fluorescence suggests the involvement of efflux mechanisms which are concentration dependent, while the dependence on temperature suggests the efflux to be a methabolic driven process. Cell suspensions were pre-loaded with dextran-FITC (70 kD, 10 µM) by exposure to extracellular pH 5.25 for 10 min. The cultures were then washed in fresh HBSS and divided into two groups, incubated at either 37°C or 4°C for four time periods (15, 60, 120 and 180 min). Results from FACS analysis are presented as fold induction relative to the fluorescent intensity of cells that were analyzed 15 min after extracellular solution was restored to pH 7.4, in terms of geometrical mean±SD (n = 9 in 3 independent experiments).

The kinetics of PIU was studied by exposing the cells’ solutions to pH values in the range of 5.0–7.4, in the presence of dextran-FITC (70 kD, 10 µM) for 60 min at 24°C (Fig. 7-A). The extent of uptake was compared to the constitutive uptake carried out at pH 7.4 (control). Decreasing the pH from 7.4 to 5.0 resulted in a non-linear and saturate-like increase of the time dependent uptake by the cells, whereas exposure of suspended cells to physiological pH 7.4, demonstrated a constitutive linear increase of dextran-FITC intensity for the entire 60 min period (R^2^ = 0.99 at pH 7.4). The range of pH<5 was not included in the data, since prolonged exposure to such low pH range inflicted some damage upon the cells and compromised the data.

Studying the rate of PIU at discrete intervals during the course of an acidic exposure was performed by a pulse labeling technique. In this experimental protocol the cells are first suspended in solution of pH 5.25 (without the presence of dextran-FITC) for different periods in the range of 0–60 min. Following the initial exposure to low pH, dextran-FITC was added to the cells’ suspension for the last set period of 10 min, after which the procedure ends and the cells were washed and prepared for FACS analysis. Thus, the extent of dextran uptake during a constant time period could be measured, as function of the exposure length of the cells to pH 5.25. Fig. 7-B reveals that the extent of PIU by the cells during 10 min treatment period, is independent of the length of the preceding incubation at pH 5.25 (P>0.05, by one-way ANOVA), suggesting that PIU rate is constant during the entire 60 min treatment.

**Figure 8:**
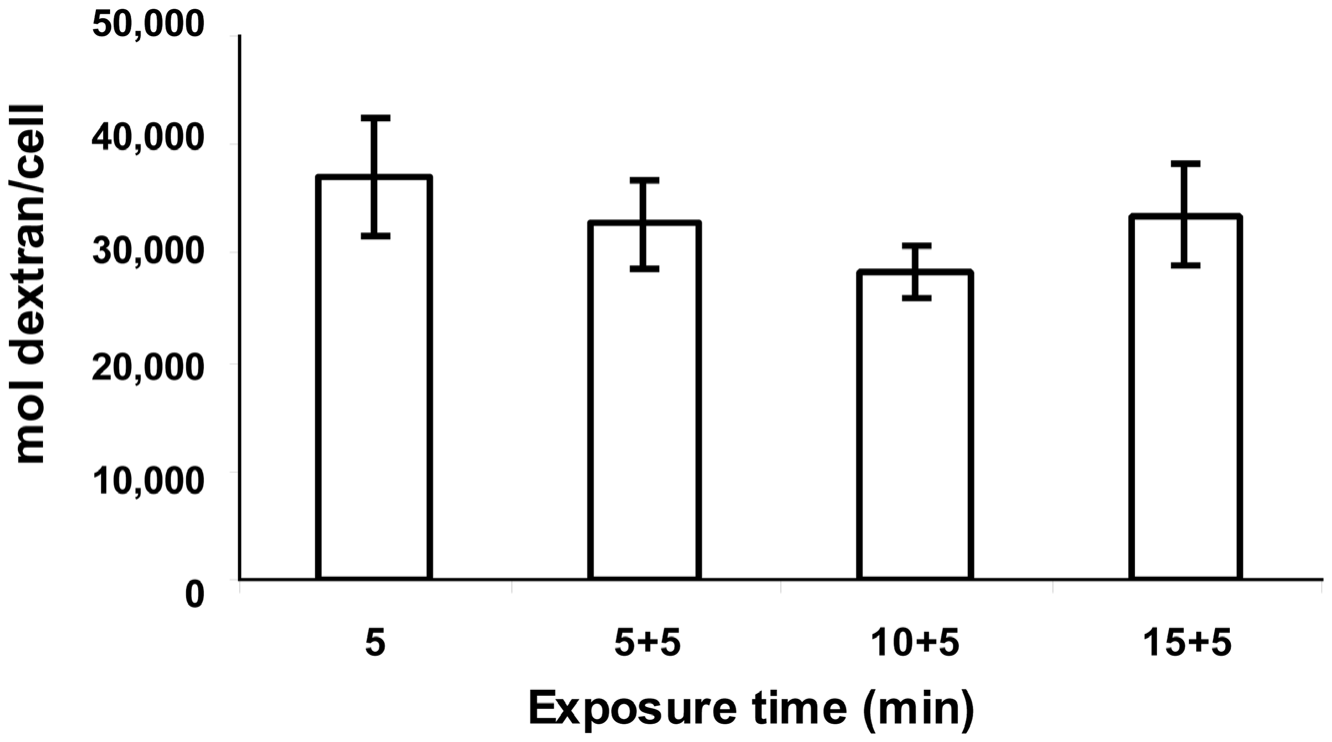
The rate of PIU as function of exposure time to low pH. HaCaT cells were harvested, suspended in HBSS and exposed to pH 5.25 in the presence of 1µM 43kDa dextran-FITC. Following treatment cells were analyzed by FACS calibrated with MESF microspheres. (A) The cells were exposed to pH 5.25 for total periods of 5, 10 15 and 20 minutes where dextran-FITC was added only for the last 5 minutes. The exposure was terminated by dilution in a large volume of cold DMEM. Results of the FACS analysis are given in terms of the number of dextran molecules per cell (mean±SD). The difference between the four groups is insignificant (P>0.05 in One-way ANOVA, n = 12).

The decline of the uptake curve, as seen in Fig. 7-A, is shown not to emanate from the entrapment of pH sensitive FITC cargo in acidic vesicles or endosomes (Fig. 5-B). The possibility that efflux processes eject dextran out of the cells was studied in cells that were first loaded with 70 kD dextran-FITC through PIU (at pH 5.25 for 10 min), followed by extensive cell wash in HBSS. The cells were then incubated at either 4°C or 37°C for periods of up to 3 hr and their fluorescence was analyzed by FACS. Fig. 7-C shows that cells incubated at 37°C, underwent an exponential decrease in the intracellular dextran-FITC content, while cells incubated at 4°C, maintained the initial dextran-FITC concentration throughout the whole incubation period without any significant decline (P>0.05, by one-way ANOVA). This temperature dependent efflux of dextran from the cells suggests the possible existence of a metabolically driven efflux. Thus the contradiction between the apparent saturation curve in Fig. 7-A and the linear uptake rate shown in Fig. 7-B, could be resolved by the removal of dextran from the cells by active cellular efflux processes. For an accurate quantification of the PIU rate during the course of an acidic exposure, we used a MESF (Molecular Equivalent Standard of Fluorescence) FACS calibration kit, in order to quantify the number of dextran molecules that enter the cell. MESF calibration curve was performed for each experimental set analyzed by FACS, enabling to calculate the number of dextran molecules per cell. By employing a pulse labeling technique, we calculated the rate of dextran uptake per minute, at discrete intervals during the acidic exposure. Cultures of HaCaT cells were harvested, suspended in HBSS and exposed to pH 5.25, for periods of 5, 10, 15 and 20 min, while Dextran-FITC (43 kD, 1 µM) was introduced to the cell suspension only for the last 5 min of the exposure period. We found that the amount of PIU, expressed in terms of dextran molecules uptake per minute, remained constant, independent of the preceding duration of exposure to low pH (Fig. 8, P>0.05, by one-way ANOVA). The constant rate of dextran uptake, as reflected from Fig. 8, enables us to calculate an uptake rate of 6,532±711 dextran molecules/cell/min (Fig. 8). If we take an average radius of ∼10 µm for suspended HaCaT cells, the intracellular dextran concentration is *∼2.8×10^+12^* mol/ml per one minute exposure. This intracellular concentration is about two orders lower than the extracellular dextran concentration used in our experiments.

**Figure 9:**
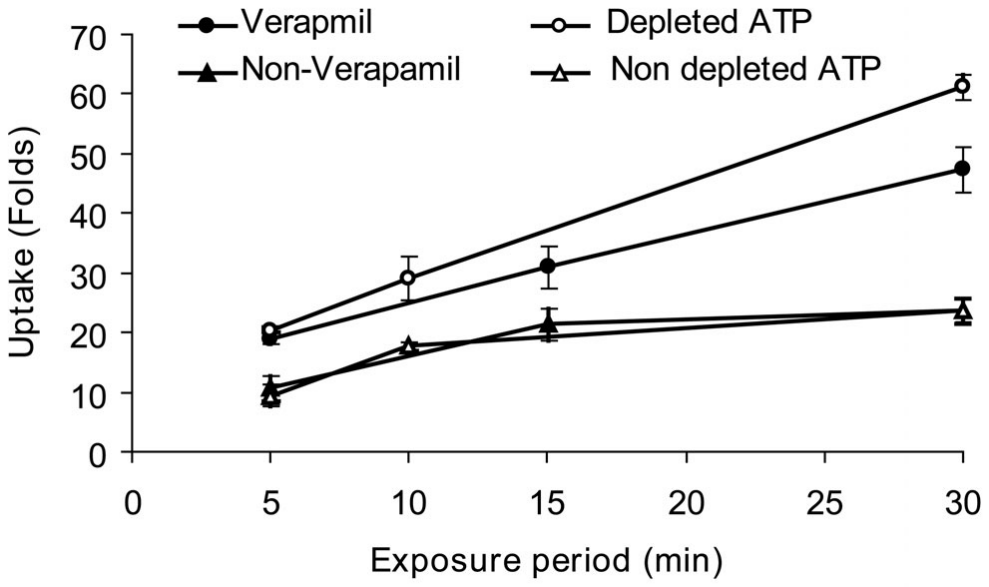
PIU as function of exposure to low pH in cells with depleted ATP or in the presence of verapamil. Suspended HaCaT cells, treated by 10 µM verapamil or depleted of their ATP pool, were exposed to extracellular pH 5.25 in the presence of dextran-FITC (70 kD, 10 µM), for 5–30 min durations. Results from FACS analyses are presented as fold induction relative to the constitutive uptake at pH 7.4 in terms of geometrical mean±SD. Linear regression of the treated cells with either verapamil or ATP depletion is R^2^ = 0.99. n = 9.

To examine the assumption that dextran is actively ejected from the cells, we attempted to decrease the activity of ATP dependent efflux pumps. We treated the cells with 20 µM verapamil, an inhibitor of the Ca^2+^ dependent ATPase activity [34], [35] for 15 minutes before treating the cells with pH 5.25 in the presence of 70 kD dextran-FITC (5 µM). The cells were treated in three exposure periods; 5, 15 or 30 min and their acquired fluorescence were analyzed by FACS (Fig. 9). Cells treated with verapamil maintain a linear correlation between cellular dextran-FITC and the length of treatment in pH 5.25 (R^2^ = 0.99), that results in higher uptake of dextran-FITC, compared to uptake in cells not treated by verapamil. Further studies regarding the dependence of dextran efflux from the cells on ATP availability were made by depleting the cellular ATP pools down to 3% of their normal level (see methods). Cells with depleted ATP pool were exposed to pH 7.4 or pH 5.25 for 5, 10 or 30 min in glucose deficient PBS and in the presence of 70 kD dextran-FITC (5 µM) and their acquired fluorescence were analyzed by FACS (Fig. 9). In cells depleted of ATP, the constitutive uptake was completely inhibited. However, in cells depleted of ATP that were exposed to pH 5.25, a linear correlation between pH 5.25 exposure length and cellular dextran-FITC concentrations was maintained (R^2^ = 0.98). Cells with depleted ATP pool acquired higher concentrations of dextran-FITC then cells with intact ATP content. Exposure durations longer then 30 min could not be accurately studied, as cells depleted of ATP begun to show signs of necrosis.

In an additional study of the relationship between ATP availability and PIU, cells were treated with oligomycin, a specific inhibitor of the F_o_ subunit in ATP syntase which halts the production of ATP in the cell [36]. The cells were treated by 10 µM oligomycin for 30 min before being subjected to 10 min treatment at pH 5.25 or pH 7.4 in the presence of 70 kD dextarn-FITC (5 µM). Control cells were treated likewise, with oligomycin replaced solely by its solvent, DMSO. Cells treated with 1% DMSO have undergone PIU to the same extent as cells not exposed to it. However, cells treated with oligomycin obtained higher extent of PIU (4.4±1.48 folds, P<0.01, by *t*-test).

## Discussion

Subjecting cells to protonation of the external surface of the plasma membrane is shown to induce the formation of membrane buds and vesicles, with a consequent enhancement in entry of macromolecules, termed PIU. The phenomenon of PIU may expand our understanding of the complex routes by which cargo is transferred across the cell membrane. Our data shows PIU to be independent of the known cellular mechanisms of endocytosis and not to involve the formation of acidic endosomes. The strata of cortical protein network underneath the plasma membrane, an important modulator of membrane dynamics [37], is known to undergo disassembly under low pH [38] and is unlikely to be a sovereign factor in PIU, as the uptake proceeds undisturbed by ATP restriction and protein kinase inhibitors (e.g. wortmanin, calyculin and genistein). Low pH activated fusion mechanism such as found in several viral proteins and toxins [9], can also be disregarded, as demonstrated by the use polysaccharide (dextran) as an uptake probe.

Protonation of the cell surface creates inward membrane structures that possess different morphology than the heterogeneous multivesicular bodies characteristic of macropinocytosis [39], yet carry visual resemblance to the caveolae (Fig. 2-A, B, and C). However, the formation of caveolae in tubular chains or grape-like clusters (Fig. 2-D, E, and F) are a feature of endothelial cells and are uncommon in epithelial cell [40]. Furthermore morphologically distinguishable caveolae are virtually absent in suspended cells [40]. We conducted our TEM studies with the epithelial keratenocyte cell-line HaCaT, held during the experiment in a suspended state rather than anchored to a substratum, thus less likely to harbor extensive caveolae. Indeed, our TEM imaging show that in cells maintained at physiological pH for 15 min after the termination of acidic conditions, we could not trace the caveolae-like structures. Caveolae are quite stable structures connected to the plasma membrane, with a departure rate of only about 2% per minute [41]. Characteristically it may take several hours before caveolin/caveolae can reappear at the plasma membrane [42]. Deportation of vesicles from the plasma membrane is expected to be accompanied by reduction in the cell area/volume ratio, which is likely to increase membrane tension. Even though the cell membrane is flaccid by nature, the loss of a large percentage of its area is bound to have an impact on its general tension and deformation resistance. It has been previously shown that an endocytic event which reduces the membrane surface area can inhibit a consequent endocytic event [43].

The finding that PIU rate remains constant for at least 60 min of exposure (Fig. 7-B and Fig. 8) implies that there is a continuous formation and recycling of membrane vesicles without substantial reduction in plasma membrane area. Taking into account the PIU’s insensitivity to caveolae inhibiting factors (e.g. phosphatase inhibitors, energy restriction and cholesterol depletion) we deduce that PIU progresses through a different mechanistic pathway than caveolae. The release of dextran from vesicles or buds into the cytoplasm (Fig. 4) could be explained by their mechanical instability due to the lack of recruitment of stabilizing coat proteins, such as clathrin or caveolin. In such an instance, the vesicles̀ membrane will remain mechanically unstable and have a thermodynamic preference for fusing into larger structures with lower membrane tension, the most prominent of which is the plasma membrane itself.

Though the rate of PIU in suspended cells appears to be constant, as reflected by our pulse labeling studies (Fig. 7-B and Fig. 8), dextran accumulation in the cells as a function of time, shows a saturation-like behavior. This saturation-like curve cannot be explained by decline in fluorescent intensity due to the intracellular entrapment of dextran-FITC in acidic vesicles. This is evident from the finding that applying the H^+^/K^+^ ionophore nigericin, had no effect on the FITC intensity of cells following PIU of dextran-FITC (Fig. 5-A2).

We would like to suggest that the saturable behavior of PIU is due to an efflux process. The efflux of dextran-FITC from the cells (Fig. 7-C) was studied by measuring the time dependent attenuation of cellular fluorescence at 37°C versus 4°C, suggesting that dextran efflux is a metabolically driven process. A better understanding of this decline in cellular fluorescence is obtained by applying inhibitors of the ATP-dependent efflux pumps (e.g. verapamil), by reducing the intracellular ATP pool either by ATP-synthase inhibitors (oligomycin) or by inhibition of the glycolysis pathway. Under these circumstances, the cellular accumulation of dextran-FITC maintains a high extent of uptake and a linear relationship as function of time (Fig. 9). These findings are explained by the inhibition of ATP-dependent efflux of dextran-FITC. One possible candidate is the ABC pump family, an important component of the cell capability to clear out foreign molecules as part of a multiple drug resistance (MDR) practice, specifically over-express in tumor cells like the Caco2/TC7 [32]. However, the ability of these pumps to remove relatively large molecules such as 70 kD dextran is still questionable.

Cellular capability to resist cytosol acidosis (Fig. S1), relies on the efficiency of proton transporters in the plasma membrane and voltage-gated proton channels [44]. The cytosolic pH data presented in Fig. S1, correlates with the findings presented in Fig. S2, which show that cells incubated in pH<5 for 10 min are beginning to suffer damage of their plasma membrane. Based on this data, we preferred to perform our experiments at pH 5.25. Long term cellular damage was rejected in viability studies performed with cells incubated in pH 5 for 2 hr (See file S1). An important damage mechanism related to cytosol acidification is mitochondrial swelling. Acidosis can increase mitochondria membrane potential and activate electrogenic K^+^ uniporters, thus shuttle K^+^ ions into the mitochondrial matrix, increase the internal osmotic pressure and produce mitochondrial swelling. This tendency is counteracted by membrane H^+^/K^+^ antiporters that extract surplus K^+^ out, preventing irreversible damage [45], [46]. However, longer period of swelling can impair the mitochondrial activity and ultimately induce apoptosis [47].

### Suggested Model for the Induction of Membrane Curvature by Surface Protonation

The phospholipids membrane elastic behavior, in terms of its spontaneous curvature, was first described by Helfrich [48]. The spontaneous curvature of the phospholipid bilayer is principally dictated by an asymmetric packing order of the phospholipids polar heads at its inner and outer monolayers (transversal asymmetry) [49]. It has also been shown that the elastic behavior of the lipid bilayer is dominated by the balance between deformation force and an opposing bending resistance and that the balance of these forces is satisfied by the deformation state defined as membrane spontaneous curvature [48]. Thus, the equilibrium shape of membrane is a result of minimization of the membrane elasto-static and electro-static energies [50]. Membrane instability may occur when repulsive forces arising from the interaction between surface charges, can overcome the stabilizing surface tension force [51]. It has been theoretically argued that for a critical value of membrane surface charged density, the membrane will spontaneously bud in the absence of any applied external force [52].

The spontaneous curvature (J_s_) of the bilayer is expressed through the spontaneous curvature of each monolayer in the couple, depending on the absolute value of the electric charge density, σ_el_, thus J_s_ =  (σ_el_^out^)^2^ – (σ_el_^in^)^2^.

When the local charge density in the outer monolayer decreases to a value much smaller than that possessed by its inner monolayer counterpart, i.e. (σ_el_^out^)^2^ << (σ_el_^in^)^2^, then the membrane spontaneous curvature adopts a negative value, forming an inward bending [53], [54]. Local decrease in the surface charge density of one membrane leaflet is expected to reduce the electrostatic repulsion among the phospholipids polar heads and consequently reduce the area occupied by each polar head, a_0_, while not affecting the packing order of the lipid tails [55]. This produces a local tension due to area asymmetry between the membrane monolayers, which can be rebalanced by a change the bilayer spontaneous curvature [49], [56], [57], [58]. For any non-zero local asymmetry of σ_el_ or a_0_ between the membrane monolayers, the bilayer will spontaneously curve toward the side possessing the higher σ_el_ and a_0_ values.

Free protons at the membrane-water interface act as counterions to the anionic polar heads of the phospholipids (Scheme S1), consequently reducing surface charge density. This effect was demonstrated in a previous study where a pH jump triggered spontaneous vesicle formation in planar dispersions of phospholipid acid (PtdOH) [59], explained by the PtdOH monolayer polar heads at the solution side becoming partially protonated and more tightly packed than the fully ionized polar-heads at the strata side [59]. The effect of protons on vesicles membranes was demonstrated by exposure of phospholipid vesicles to localized pulses of low pH, which produced the formation of inward tubular membrane invaginations [60]. It should be pointed out that the effect of protons could not be reproduced with other monovalent cations (e.g. sodium or potassium) at equivalent concentrations. Protons possess higher affinity towards the phospholipid polar heads compared to all other mono and divalent cations, as a result of their higher electrostatic potential energy, due to their small atomic radii [61].

The induction of a local membrane spontaneous curvature in a direction opposite to the general steady-state shape, introduces tension into the system, coupled by higher deformation energy. Indeed, in studies performed with phospholipids vesicles, membrane tension and bending rigidity were found to increase with the higher proton concentrations in the external solution. Maximum vesicular membrane tension was reached toward the membrane isoelectric point at pH 4 [62], [63], [64]. Our data reveals that the plot of uptake as a function of the external pH (Fig. 6-A) takes a sigmoid-like pattern with a steep rise of uptake in the range 4> pH>3. This pH range coincides with the isoelectric point for the ζ potential of phospholipids vesicles, measured as function of the external pH [64], [65]. We therefore suggest that the results presented in Fig. 6-A can be rationalized by the rate of PIU being dependent on the proton induced attenuation of surface charge on the external leaflet of the plasma membrane. It is important to note that although the major contributing constituents to the cell’s ζ potential are the charged glycoproteins and glycolipids of the glycocalyx coat, theoretical modeling [66] and experimental measurements [67], [68] have shown that the glycocalyx offers no significant buffering effect to the membrane surface from the bulk pH. This is in line with our observation that the enzymatic degradation of the glycocalyx has no effect on the extent of PIU mediated uptake (Fig. S3). It should be pointed out that proton diffusion in the bulk medium is slower than proton spreading over the membrane, as the last one is facilitated by the hydrogen-bonded networks at the surface (26). Thus, a higher proton concentration at the surface is maintained relative to the bulk solution (28, 29).

The deformation of planar membrane into bud structure is a local event. It is widely accepted that during an endocytic process, this event is driven by specialized proteins that apply mechanical constraints to the membrane surface forcing the membrane to bend [3]. This is accomplished by a direct insertion of protein domains into the lipid bilayer, changing the structure of one with respect to the other or by modifying the local lipid compositions [5]. The forces that determine the bilayer susceptibility to further undergo fission and produce endosomes, come from proteins and in most cases from membrane-bound cytoskeletal proteins [37]. Within the phenomena of PIU, the localization of membrane deformation (demonstrated in Fig. 1 to occur at discrete membrane sites and not randomly distributed over the cell surface) is attributed to the lateral non-uniform distribution of molecular components in the plasma membrane, creating microdomains, varied by their lipid composition, density and charge. Microdomains in the plasma membrane respond differently to membrane tension or produce different asymmetric imbalance under external low pH. For example, membrane domains rich with cholesterol or sphingomyelin (i.e. lipid rafts) were shown to possess different bending module and curvature than their surrounding phospholipid bilayer [69], [70]. Lipid rafts are thicker than the surrounding membrane [71], [72] and therefore to avoid exposure of hydrophobic tails to water at the raft boundary, monolayers should elastically deform. Deformation energy per boundary length defines the line tension between the elastic moduli of the raft and that of the surrounding membrane. Line tension is greater when raft curvature and spontaneous curvature have the same sign (i.e. curving in the same direction) and is smaller when they have opposite signs [73]. Thus, the membrane within the boundary should tend to curve in an opposite direction to the spontaneous curvature of the plasma membrane. Locally, a large domain has a larger boundary length which means an increase in local line energy. Hence, line energy can effectively be reduced if membrane within the boundary bulges out of plane to form vesicles, effectively shortening its boundary length with the surrounding membrane [74]. When the surface area of a bud grows, the neck will constrict at the lipid phase interface so that the bending energy of the bud will be distributed over a larger area with smaller curvature. Hence the growth of the bud enforces the narrowing of its neck diameter [75]. Vesicles fission from bilayer membranes do not usually occur spontaneously since the repulsive energy between two approaching membranes is very high at atomic distances. Promoting membrane fusion is helped by negative curvature stresses and the formation of non-bilayer fusion intermediates called membrane stalks, facilitated by lipids of high spontaneous [76]. At separation distance as small as several nanometers between two leaflets of the membrane, thermal fluctuations are sufficient to fuse the leaflets and pinch off the bud from the membrane tubule [77], [78].

### Concluding Remarks

This study demonstrates that exposure of the cell surface to a high concentration of protons is associated with enhanced uptake. This uptake appears to proceed through proton-induced formation of inward invaginations of the plasma membrane with consequent vesiculation. These vesicles undergo fast recycling accompanied by the release of their content into the cytosol. We suggest that this uptake proceeds through a novel pathway, as it is unaffected by conditions and agents that inhibit endocytosis and is unrelated to fusion proteins, that once triggered by low pH enable membrane penetration of toxins and viruses.

We propose that cell membrane mechanical properties are determinant in the dynamics of proton-induced uptake. To deform the membrane into the tubular or vesicular shapes, the membrane elastic resistance forces must be balanced by some additional forces. We suggest that these forces are generated by an enhanced asymmetry of local cross-membrane surface charge densities. We would like to advance the idea that while the employment of specialized proteins in endocytosis confers superior control and precision to the process, there could be an additional mechanism that, to some extent, progress without them.

By inducing low pH microenvironment in-vivo, one can envision the localized delivery of various therapeutic modalities into cells. Such efficient cross-membrane delivery of drugs can permit the deployment of relatively low dosage therapy thus reducing the accompanied adverse effects.

## Materials and Methods

### Materials

K^+^PBS (130 mM potassium buffer): CaCl_2_ 0.01 gr/L, MgCl_2_*6H_2_O 0.1 gr/L, Na_2_HPO_4_ 1.148 gr/L, KH_2_PO_4_*H_2_O 0.2 gr/L, NaCl 0.58 gr/L, KCl 9.7 gr/L, dissolved in deionized water. Karnovsky (x2 stock solution): 6% paraformaldehyde, 1% glutaraldehyde in 0.2M cacodylate buffer. Nigericin, Staurosporine, Dextran-FITC 70 kD, MES (2-(N-morpholino)ethanesulfonic acid), HCl (hydrochloric acid 32%), and PI (propidium iodide), were purchased from Sigma-Aldrich, Rehovot, Israel. BCECF-AM (2′,7′-bis-(2-carboxyethyl)-5-(and-6)-carboxyfluorescein-acetoxymethyl ester) were purchased from (Invitrogen, USA).

#### Cell culture

PBS (phosphate buffered saline), PBS (Ca^++^ and Mg^++^ free), DMEM (Dulbecco’s Modified Eagle Medium, 4.5 mg/ml glucose), RPMI 1640 (Roswell Park Memorial Institute) culture media, HBSS (Henk’s balanced salt solution), FCS (fetal calf serum), trypsin solution (0.25% with 0.05% EDTA), PSN (penicillin 10,000 unit/ml, streptomycin 10mg/ml, nistatin 1250 unit/ml) L-glutamine solution (200 mM), Non-essential amino acid solution, HEPES (4-(2-hydroxyethyl)-1-piperazineethanesulfonic acid, 1M) and Tryphan-blue (0.4%), were purchased from Biological Industries (Beit Ha’emek, Israel). Fibroblast-like monkey kidney cells (COS-7, ATCC No. CRL-1651), human keratenocytes (HaCaT [31]) and human intestinal goblet cells (HT29-mtx [79], kind gift of T. Lesuffleur, INSERM, Paris, France) were cultured in DMEM, supplemented with 2 mM L-glutamine, 10% FCS and 0.2% PSN solution. Human intestinal entherocytes (Caco2-TC7 [80], kind gift of M. Rousset, INSERM, Paris, France) were culture in DMEM, supplemented with 2 mM L-glutamine, 20% FCS, 0.2% PSN solution and 1% non-essential amino acids. Lymphoblast cells TK6 (ATCC No. CRL-8015) were cultured in suspension in RPMI supplemented with 16.5% FCS and 1% PSN. All cells were grown at 37°C, in a humid atmosphere of 5% CO_2_ in air. Cells were harvested before reaching ∼80% confluence by employing trypsin solution for 5 min at RT. The harvested cells were centrifuged for 2 min at 400 g. The supernatant was aspirated and the cell pellet was re-suspended in fresh growth media.

#### Uptake studies

For the studies of adherent cultures, cells were seeded on surface treated 24 well plates and incubated in growth medium at 37°C humid atmosphere with 5% CO_2_. Experiments were performed when cells reached approximate confluence (∼5×10^5^ cell/well). Culture wells were washed twice with HBSS before being subjected to the experimental procedure. Lowering the pH of HBSS was carried out with 10 mM MES and a titer of hydrochloric acid. During exposure, each well contained 250 µl solution volumes and stopping the acidic exposure was accomplished by adding 1 ml of cold DMEM into the wells, thus recovering the physiological pH of 7.4. Experiments were planned and conducted in a manner that enables all wells in a single plate to enter the washing step at the same time. The wells were washed twice with cold PBS and the cells were harvested by 10 min incubation with 0.25 ml trypsin solution and 0.25 ml PBS (deprived of Ca^2+^ and Mg^2+^) at room temperature. The harvested cells from each well were transferred to a 5 ml tube containing 1 ml cold DMEM with 10% FCS. These tubes were centrifuged for 2 min at 400g, the supernatant aspirated and the cells pellets were re-suspended in 0.5 ml cold HBSS. Immediately before FACS analysis, we added to each tube 0.01% trypan-blue (TB), which served both for quenching the fluorescence of extracellular FITC [17] and for staining necrotic cells with compromised membranes. In cells subjected to solutions of low pH in the presence of dextran-FITC, TB reduces cell fluorescence in flow cytometry by 20%. In studies of cells in suspensions, harvesting the cells was performed before they were subjected to the treatment protocol.

#### ATP depletion

The cells were washed with PBS twice and were incubated with 6 mM Iodoacetamide, 0.01% azid and 10 mM Inosine for one hour at 37°C. Cellular ATP was determined using a luciferin-luciferase luminescence assay (ATPlite, PerkinElmer, USA).

#### Flow cytometry (FACS)

Flow cytometry analysis was carried out with FACScalibur (Becton@Dickson, San Jose, CA), employing a 488 nm argon laser excitation. FITC fluorescence was detected by 530/30 nm filter (FL1), PI fluorescence was detected by 580/30 nm filter (FL2) and the fluorescence of TB was detected by a 680/30 nm filter (FL3). 10,000 cells were collected from each sample and analysis of data was performed using cyflogic 1.2.1 (CyFlo LTD, Finland) application software. To eliminate signals due to cellular fragments, only those events with forward and side scatter comparable to untreated cells were analyzed. Cells labeled by TB were considered dead and rejected from analysis. Quantum FITC MESF kit (Bangs Labs, USA) was used in the quantification of FITC fluorescence intensity in units of Molecules of Equivalent Soluble Fluorochrome (MESF). When establishing a calibration plot, no further adjustments of the instrument settings has being done (e.g. amplifier gains, PMT voltages, etc).

#### Fluorescent Microscopy

For acquiring microscopic images we employed a Scanning Confocal Laser Microscope (SCLM; LSM 410, Zeiss, Germany) or a fluorescent microscope (Axio-observer Z1, Zeiss, Germany). In SCLM, computer-generated images of 0.5 µm optical sections were obtained at the approximate geometric center of the cell as determined by repeated optical sections. For acquiring images of surface adherent cells, cultures were grown on glass cover slips coated with 1% gelatin, or in black 96 well-plates with thin glass bottom. Cell cultures were optically analyzed live or following fixation with 4% paraformaldehyde. Digital images were acquired through high definition gray scale camera and processed by axiovision software (Zeiss, Germany). Annotation was made using Illustrator CS software (Abode, USA).

#### Transmission electron microscopy (TEM)

Cells were harvested and suspended in HBSS, before being subjected to the experimental treatments. The cells were incubated in MES buffered saline (pH 5) for the specified duration which was terminated by direct addition of 1∶1 volumes of cold ×2 karnovsky fixative to the cells’ suspension for additional 20 min. The suspended cells were sedimented at 3000 g for 5 min and a subsequent 12,000 g for 5 min. The cells were post fixed in 1% OsO_4_ in PBS for 2 hours at 4°C, followed by dehydration in graded ethanol and embeded in Glycid ether. Thin sections were mounted on Formvar/Carbon coated grids and stained with Uranyl acetate and Lead citrate at room temperature. Digital image acquisition was performed by a Jeol 1200EX transmission electron microscope (Jeol, Japan). Annotation was made using Illustrator CS software (Abode, USA).

#### Determination of intracellular pH

BCECF-AM is a non-fluorescent, cell permeable molecule that is transformed by intracellular esterases into the charged, non-permeable and fluorescent BCECF molecule. Cell loading with 10 µM BCECF-AM was performed by incubating the cells for 30 min in HBSS at 37°C. Following the incubation period, the cells were centrifuged at 400 g for 1 min, the solution aspirated, and cells were re-suspended in HBSS. For analysis of intracellular pH, the cells were transferred to 96 well microplates and their excitation ratio was calculated from the fluorescent emission intensities acquired at Ex 430 nm/Em 535 nm and Ex 490 nm/Em 535 nm. The pH-dependent spectral shifts exhibited by BCECF allow calibration of the pH response in terms of the ratio of fluorescence emission intensity at 535 nm when using two different excitation wavelengths λ1 = 490 nm and λ2 = 440 nm [81]. Calibration curve for BCECF excitation ratio in terms of intracellular pH values, was made with cells pre-loaded with BCECF and suspended with 10 µM nigericin in K^+^PBS at 5 different pH values in the range of 7.4> pH>5.4.

#### Statistics

Results were collected from several independent experiments and statistical analyses was performed using two tails student t-test or one-way ANOVA, with the null hypothesis rejected for a probability value of P<0.05. Statistical analysis was performed using Microsoft Excel spreadsheets.

## Supporting Information

Figure S1 **Intracellular pH as function of incubation time in low pH solution**. HaCaT cells, loaded with the pH fluorescent probe BCEFC, were suspended in MES-HBSS solutions at 37°C and the extracellular pH was altered by titration with concentrated HCl. The cytosolic pH level was determined from BCECF fluorescent intensity using the ratiometric method in 12 independent measurements.
(TIF)

Figure S2 **Fraction of cells stained with PI as function of external pH.** The staining of a cell nucleus with PI indicates a compromised plasma membrane integrity and is considered a reliable sign of necrosis. Cells (HaCaT, Caco-2/TC7 and TK6) were incubated in MES-HBSS solution of different pH, at 37°C for 10 min and analyzed by FACS in the presence of PI (25 µg/ml). The fraction of necrotic HaCaT, TK6 and Caco-2/TC7 cells in the pH range of 7.4 to 5 is not significantly different (P>0.05, ANOVA), n = 9 for each cell line.
(TIF)

Figure S3 **Enzymatic degradation of the glycocalyx does not inhibit PIU**. The uptake of dextran-FITC was measured in cells after their treatment with enzymatic solution that cleaves surface glycocalyx. Enzyme treated cells and intact untreated cells were exposed to pH 5.3 in the presence of dextran-FITC and their fluorescence was analyzed by FACS. The results are presented as folds of geometrical mean±SD of FITC fluorescence intensity relative to intact cells, in two independent experiments (P>0.1, one-way ANOVA), n = 12.
(TIF)

Scheme S1 **Model of proton induced cell membrane budding.** High concentration of protons at the external surface of the cell membrane acts as counterions to the phospholipids anionic polar-heads and reduces their electric charge. Reduced electrostatic repulsion permits the polar-heads to occupy a smaller area and consequently deform the membrane curvature (a). In the presence of a membrane tension line (e.g. at the boundary of lipid rafts), this curvature may develops into invagination (b) and will further bud (c).
(TIF)

File S1 (DOC)
