# Supplementary figures and images for: Enhancement of Cell Membrane Invaginations, Vesiculation and Uptake of Macromolecules by Protonation of the Cell Surface
Source: PLoS One. 2012 Apr 30;7(4):e35204. doi: 10.1371/journal.pone.0035204 (PMC3340387; doi:10.1371/journal.pone.0035204)

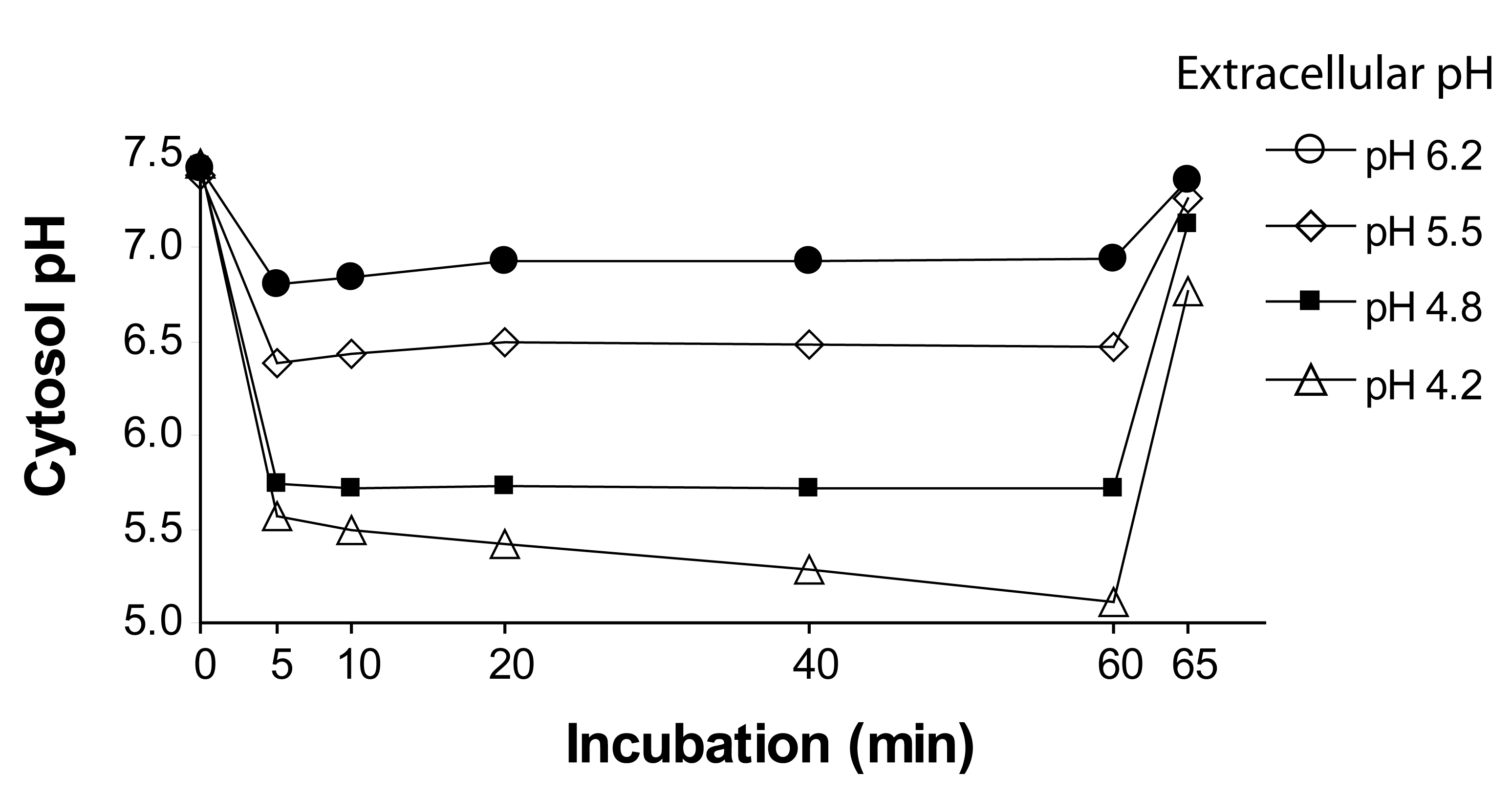

Supplement: Figure S1 — Intracellular pH as function of incubation time in low pH solution. HaCaT cells, loaded with the pH fluorescent probe BCEFC, were suspended in MES-HBSS solutions at 37°C and the extracellular pH was altered by titration with concentrated HCl. The cytosolic pH level was determined from BCECF fluorescent intensity using the ratiometric method in 12 independent measurements. (TIF) [file pone.0035204.s001.tif]

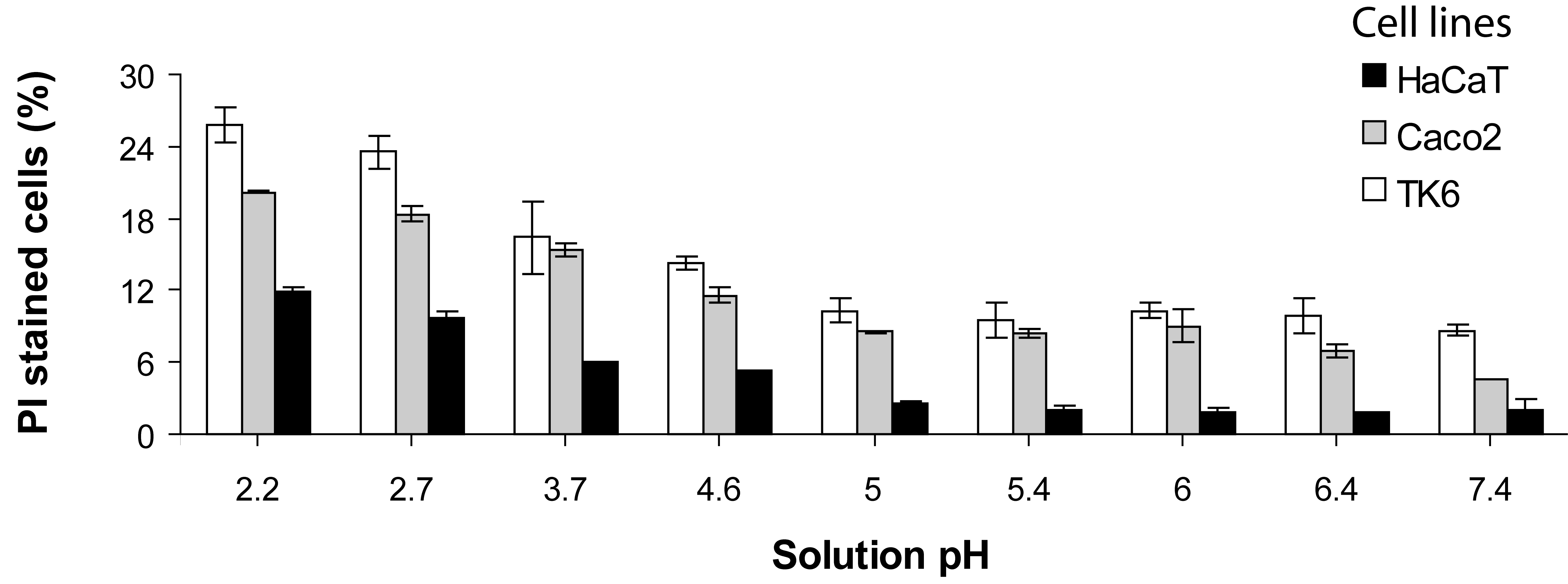

Supplement: Figure S2 — Fraction of cells stained with PI as function of external pH. The staining of a cell nucleus with PI indicates a compromised plasma membrane integrity and is considered a reliable sign of necrosis. Cells (HaCaT, Caco-2/TC7 and TK6) were incubated in MES-HBSS solution of different pH, at 37°C for 10 min and analyzed by FACS in the presence of PI (25 µg/ml). The fraction of necrotic HaCaT, TK6 and Caco-2/TC7 cells in the pH range of 7.4 to 5 is not significantly different (P>0.05, ANOVA), n = 9 for each cell line. (TIF) [file pone.0035204.s002.tif]

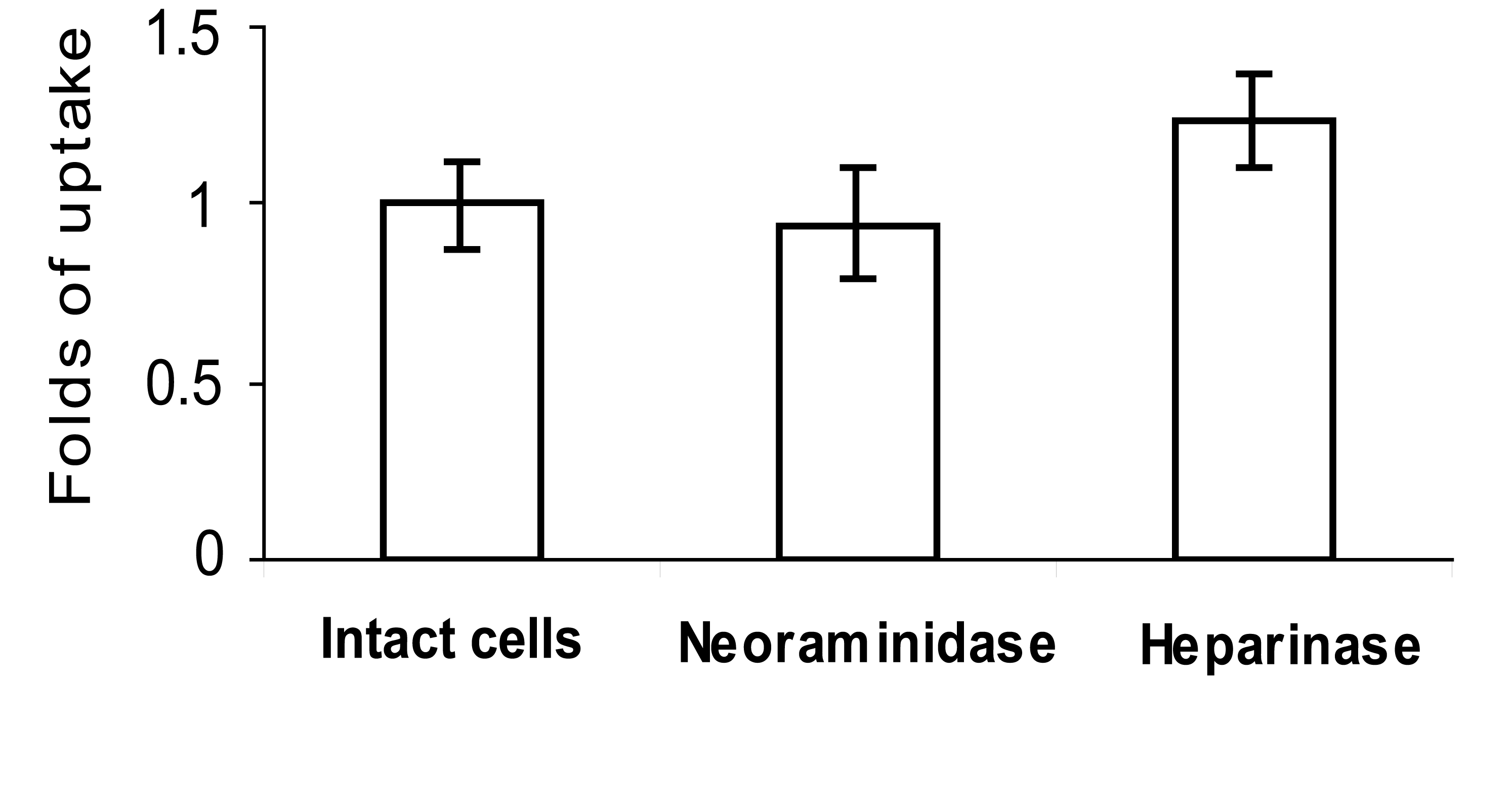

Supplement: Figure S3 — Enzymatic degradation of the glycocalyx does not inhibit PIU. The uptake of dextran-FITC was measured in cells after their treatment with enzymatic solution that cleaves surface glycocalyx. Enzyme treated cells and intact untreated cells were exposed to pH 5.3 in the presence of dextran-FITC and their fluorescence was analyzed by FACS. The results are presented as folds of geometrical mean±SD of FITC fluorescence intensity relative to intact cells, in two independent experiments (P>0.1, one-way ANOVA), n = 12. (TIF) [file pone.0035204.s003.tif]

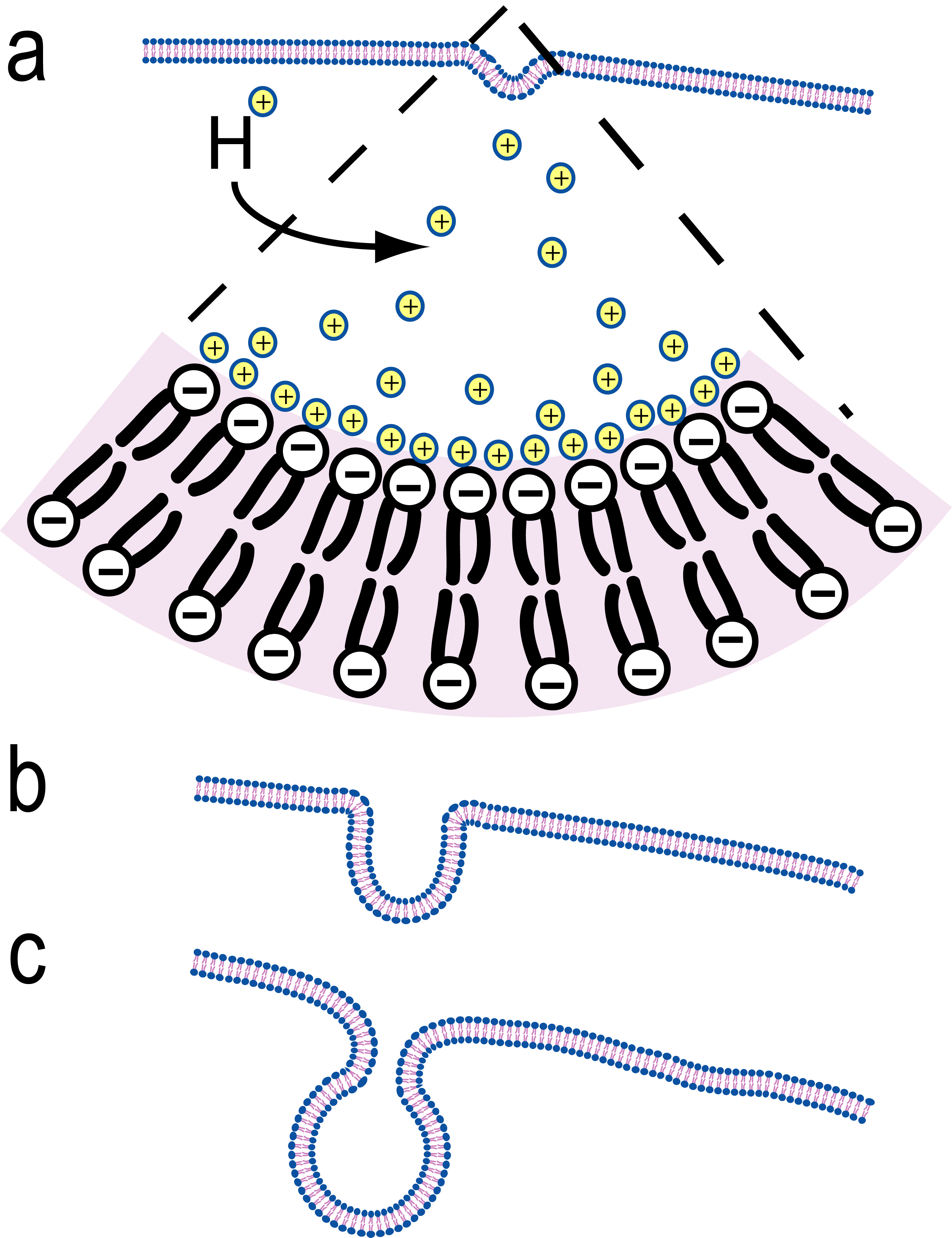

Supplement: Scheme S1 — Model of proton induced cell membrane budding. High concentration of protons at the external surface of the cell membrane acts as counterions to the phospholipids anionic polar-heads and reduces their electric charge. Reduced electrostatic repulsion permits the polar-heads to occupy a smaller area and consequently deform the membrane curvature (a). In the presence of a membrane tension line (e.g. at the boundary of lipid rafts), this curvature may develops into invagination (b) and will further bud (c). (TIF) [file pone.0035204.s004.tif]
