# Supplementary material for: Enhancement of Cell Membrane Invaginations, Vesiculation and Uptake of Macromolecules by Protonation of the Cell Surface
Source: PLoS One. 2012 Apr 30;7(4):e35204. doi: 10.1371/journal.pone.0035204 (PMC3340387; doi:10.1371/journal.pone.0035204)
Supplement: File S1 — (DOC) [file pone.0035204.s005.doc]

***Supporting information***

**Cell Viability is not impaired by short exposure to external pH 5.**

Apoptosis: Caco-2/TC7 or HaCaT cells were exposed to pH 5 for a period of two hours and compared to the to the apoptotic effect induced by staurosporine (as a positive control). Two hours after the external pH was restored to its physiological value of pH 7.4, annexin-FITC binding to the outer membrane leaflet was measured by flow cytometry. There was no increase in the binding of annexin to cells exposed to pH 5 as compared to cells exposed to pH 7.4.

Viability: Viability assays were performed 24 hours after terminating the exposure of HaCaT cells to an acidic environment. The assessment included alamar blue assay (for cellular enzymatic metabolism), crystal violet assay (for the total number of nuclei) and BRdU assay (for DNA synthesis). In all three assays, no significant difference was found between cells exposed to pH 5 and cells exposed to pH 7.4.

**SI Methods**

**Enzymatic degradation of the glycocalyx.** Cells were harvested using trypsinization, washed and treated with enzymatic solution of either neuraminidase or heparinase mixture (heparinase I, 14.5 u/ml; heparinase II, 2.4 u/ml; heparinase III, 1.35 u/ml. Sigma-Aldrich, Rechovot, Israel) for two hours. The cells were exposed for 10 minutes to 70 kD dextran-FITC solution (5 µM) in HBSS at pH 7.4 or pH 5.3, washed and prepared for flow cytometry as described earlier.

**Annexin assay for induction of apoptosis.** Untreated cells were exposed to the apoptotic agent staurosporine for obtaining a positive control. Cells were centrifuged (1 min at 400g) and re-suspended in a 500 µl annexin-FITC buffer solution (Annexin-V kit, Biovision, USA). 5 µl Annexin V-FITC was added to the cells, followed by 5 µl PI for 15 minutes incubation before the cells were analyzed by flow cytometry.

**Alamar blue viability assay.** Cell cultures were grown in transparent 96 well-plates. Following their exposure to low pH, the wells were washed and incubated for 1 hr or 24 hr in growth medium (37°C, 5% CO_2_). At the end of the incubation period, the wells were washed twice in PBS, refilled with 90 µl DMEM (phenol red free), 10% FCS, 10 µl alamar-blue solution (Serotec, U.K.) and incubated for 60 min. Fluorescence was monitored at 485 nm/959 nm in a multiplate reader (GENius, Tecan, Austria).

**BRdU incorporation assay.** Cell proliferation was assayed using Chemiluminescent Cell Proliferation ELISA BrdU assay kit (Roche, Germany). Cells are grown n flat bottom white microplates where they have undergone the experimental treatment. Following treatment, BrdU labeling solution was added per well for 24 hr incubation (5% CO_2_ humid atmosphere at 37°C). Next, the wells were washed and incubated for 30 min with a fixation solution before being washed again. Anti-BrdU solution is added to the wells for 60 min, followed by extensive washing and incubation with the substrate solution for 10 minutes, before measuring the luminescence.

**Crystal violet nuclear assay.** Cell cultures were grown in transparent 96 well-plates. Following their exposure to low pH, the wells were washed and incubated for additional 24 hr in DMEM (5% CO_2_ humid atmosphere at 37°C). At the end of the incubation period, the cells were washed twice in PBS, fixated with 4% paraformaldehyde for 10 min and washed twice in PBS. Then the cells were stained with crystal violet (0.5%) for 10 min, followed by 5 repeated washings with PBS. For extracting the dye, cells were incubated for 15 min with 100 µl PBS containing 1% SDS then supplemented with additional 100 µl PBS. Light absorption was measured at 560 nm in a multiplate reader (GENius, Tecan, Austria).
